# Braiding Thermoplastic and Glass Fibers in Composite Dental Post Improves Their Mechanical Compatibility, In Vitro Experiment

**DOI:** 10.3390/ma14092294

**Published:** 2021-04-29

**Authors:** Esraa M. Abdelkader, Khaled Nassar, Juan Melchor, Guillermo Rus

**Affiliations:** 1 Department of Structural Mechanics, University of Granada, 18071 Granada, Spain; grus@ugr.es; 2 Department of Textile, Faculty of Applied Arts, Helwan University, Cairo 11795, Egypt; khaled.mansour@buc.edu.eg; 3 Department of Textile, Faculty of Applied Arts, Badr University in Cairo, Cairo 11829, Egypt; 4 Biomechanics Group (TEC-12), Instituto de Investigación Biosanitaria, ibs.GRANADA, 18012 Granada, Spain; jmelchor@ugr.es; 5 Excellence Research Unit “ModelingNature” MNat, University of Granada, 18071 Granada, Spain; 6 Department of Statistics and Operations Research, University of Granada, 18071 Granada, Spain

**Keywords:** dental materials, root canal posts, fiber-reinforced composites (FRCs), young’s modulus, shear modulus, fiber volume fraction (FVF), endodontic

## Abstract

Mechanical compatibility with the human dentin is a considerable issue when fabricating dental fiber posts. To this purpose, this study introduces a new method of fabricating compatible dental posts using braiding techniques of thermoplastic fibers (matrix) with glass fibers (reinforcement). Fifty fiber-reinforced composite (FRC) posts of thermoplastic yarns polypropylene (PP) braided with continuous filaments glass fibers (GFs) for reinforcement, varying in fiber volume fraction (FVF), and core types are fabricated and tested. Posts are performed using a braiding machine, and braids are placed in an aluminum mold. The filled mold is playced inside an oven at the melting temperature of the polypropylene to produce the final post’s shape. An ultrasonic test is conducted to measure the shear modulus and Young’s modulus of FRC post specimens by measuring the velocities of both the P-wave and S-wave. In order to ensure the accuracy of the measurements, each sample is measured three times, and then the means and standard deviations of each sample are calculated before analyzing the test results using the means of two steps, namely, clustering and comparing the P and R² values of each cluster, which revealed that FVF, fiber mass, and core type of the specimen had a significant effect on the resulted Young’s and shear modulus. The results indicate that the proposed method can fabricate competitive dental posts with regard to different fabricating variables. The samples show Young’s modulus ranges of from 10.08 GPa to 31.83 GPa. The following tested hypothesis is supported: the braiding technique of thermoplastic fibers with glass fibers will improve the mechanical compatibility of the resulting posts (ex vivo).

## 1. Introduction

Fiber-reinforced composites (FRCs) have been introduced as a superior alternative to many conventional materials over the past two decades because of their enhanced structural and mechanical properties [1,2,3,4]. An FRC is the combination of two or more different substances; one of them is introduced in the matrix phase while the other materials comprise particles or fibers in order to give the composite materials their unique characteristics and reinforce them [5,6,7,8].

FRC posts are among the most important composite applications in the dental field, and they have been widely used for endodontically treated teeth. The main advantage of using FRC posts is their essential role in preventing many of the vertical root fracture incidents; this advantage can be attributed to the Young’s modulus of the fiber-reinforced posts, which is considerably similar to that of the natural tooth’s dentin (18 GPa) [9,10]. Another advantage of using the FRC posts is their aesthetic superiority to metal posts [11,12].

Moreover, metal posts are known to result in some allergies, while using FRC posts has not been observed to cause allergic reactions or infections [13]. Due to the different advantages of the FRC represented in the better mechanical properties of composites, they have been used more frequently as root canal posts for the core of endodontically treated teeth [14].

Continuous fiber composite materials have been demonstrated as cutting edge structural materials with applications in different fields, such as in the aviation industry, infrastructure, and dental materials (e.g., dental posts) [15]. The distinguishing features of the FRC are their unique and functional properties, which enable them to contribute to various applications [16].

FRCs were previously fabricated using a resin of thermoset emulsions with the reinforcement fibers embedded in these emulsions in a conventional method of fabricating composite materials, or, as in other traditional manufacturing processes, the fibers’ placement was determined with the use of sewing or tape systems [17,18].

In the novel method suggested in this paper, resin (matrix) is used in a form of thermoplastic yarns to utilize its different advantages, such as low melting points, ease of formation, and low prices compared to thermosets.

Thermosets and thermoplastics are two different forms of polymer substances that are differentiated based on their reaction when applying heat to them. The essential distinction between the two substances (thermoset and thermoplastic) is that the thermoset becomes more reinforced when exposed to high temperature; however, it cannot be remolded later into the very initial shape, whereas thermoplastics can be exposed to both high and low temperatures and consequently can be remolded several times after the first time without having any chemical changes in their initial properties [19]. However, reaching the glass transition temperature, both thermosets and thermoplastics can deform dramatically according to their crystalline-to-amorphous structure ratios [20,21,22].

The current investigation is a part of the on-going research on fabricating dental fiber posts, which, in brief, are composite rods with a 1.5:2.2 mm diameter. The fibers used in this study are thermoplastic polypropylene yarns and glass fibers yarns. A hybrid of braided thermoplastic fibers and unidirectional glass fiber is selected as the fabricated posts to satisfy the stiffness requirements of the intended application.

Accordingly, this study develops a new method of fabricating dental fiber posts based on fiber-reinforced composite properties [23]. The fabricating process discussed here was conducted using braiding techniques, while the composite forming process was achieved through the melting process.

The aim of this study was to test the following conceptual hypotheses (objectives):

**Hypothesis** **1.** *The braiding procedure could provide a good solution for fabricating dental fiber posts.*

**Hypothesis** **2.** *Thermoplastic filaments could be used as a matrix of reinforced composites used for the ex vivo application of dental fiber posts.*

**Hypothesis** **3.** *The fiber volume fraction is directly proportional to the final post modulus.*

**Hypothesis** **4.** *Fiber material native Young’s modulus is correlated with the post Young’s modulus and shear modulus.*

This paper is divided into several sections: Firstly, a brief introduction to the content is provided. The second section discusses the materials and detailed methodologies used in this work. An experimental approach is employed to test the new method of fabricating dental posts. Thirdly, the results of the ultrasonic tests for the fabricated posts are compared using the means of two-steps clustering, descriptive analysis, and a Pearson’s correlation study. Finally, sections regarding the discussion of the results and the conclusion of the work are presented.

## 2. Materials and Methods

### 2.1. Materials

The materials used in the research project can be divided into two categories.

#### 2.1.1. Category One: Reinforcement Fibers

Different types of fiberglass yarns were used as glass fibers are considered the dominant type of reinforcement in the composite industry simply because of their versatility and excellent performance-to-price value, which means that it is much cheaper than carbon. Glass fibers are available in different grades: E, S, and C, where E is used for high modulus, S for high strength, and C for chemical resistance.

The fiberglass yarns were divided into two main types according to their silane: The first type was E-glass fiber treated with starch (ECE225) with a count of “22 Tex”. The second type was divided into two classes of glass fiber with a specialized silane for coupling with thermoplastic fibers under the commercial name of “561 sizing”: 1—E-glass class (ECDE75) with a count of “66 Tex”; 2—S-glass class (SCG75) with a count of “68 Tex”.

Fiberglass yarns were generously donated by AGY industries, located in the Aiken, SC, USA. The different yarn specs are listed in Table 1, Table 2 and Table 3.

#### 2.1.2. Category Two: Thermoplastic Fibers (Matrix)

Thermoplastic polypropylene (PP) yarns were used; the count of 300 Denier was constant regardless of whether plied or single yarns were used. The technical specs and physical properties of polypropylene yarn are listed in Table 4. Thermoplastic yarn in different braids acts as a homogeneous and well-distributed matrix after the melting stage.

### 2.2. Methods

The fabrication process within the current work was accomplished through using braiding techniques to braid both polypropylene (PP) yarns and glass fiber (GF) ones, while composite formation was achieved using the melting process. Thermoplastic polypropylene yarns were melted in order to act as a composite matrix, while the glass fiber yarns remained as the reinforcement quantum.

A YITAI braiding machine (Xiamen Yitai Industrial Co., Ltd, Fujian, China)was used to perform the required braids for the dental fiber post; the braiding machine has 48 spindles. In this machine, a digital speedometer is used to control the braiding spindles’ speed; in our case, this was set to 25 m/h (±5). Two gears of 42 and 20 teeth were meshed to provide the required speed ratio between the take-up speed and the spindle speed and, thus, provide an acute braiding and constant angle of almost 40 degrees.

For the present study, the essential parameters that the control braiding structure during the braiding process were as follows: the different yarn counts, the distribution of these yarns all over the machine spindles, the braiding spindle speed, and the take-up speed. It is the ratio between spindle speed and machine take-up speed that characterizes the braiding angle [25]. This ratio was kept steady during the whole braiding process and subsequently produced a preform of the constant braiding angle.

Pure axial glass fiber, pure axial thermoplastic, and mixed axial thermoplastic with glass fiber yarns were introduced into the center of the circular braid to form three different cores (core GF: 100% glass fiber–0% polypropylene; core PP: 0% glass fiber–100% polypropylene; core mix: 50% glass fiber–50% polypropylene).

Different setups were made using different numbers of working spindles from the machine’s available 48 spindles. However, because of the geometry limitations of yarn jamming, the braiding angle that can be achieved for a particular braided preform is controlled by factors such as the number of actual working spindles, the yarn count, and the required final diameter of the braided perform [26]. Thus, almost one-third of the spindles’ capacity was used to produce the fiber post samples.

**Figure 1:**
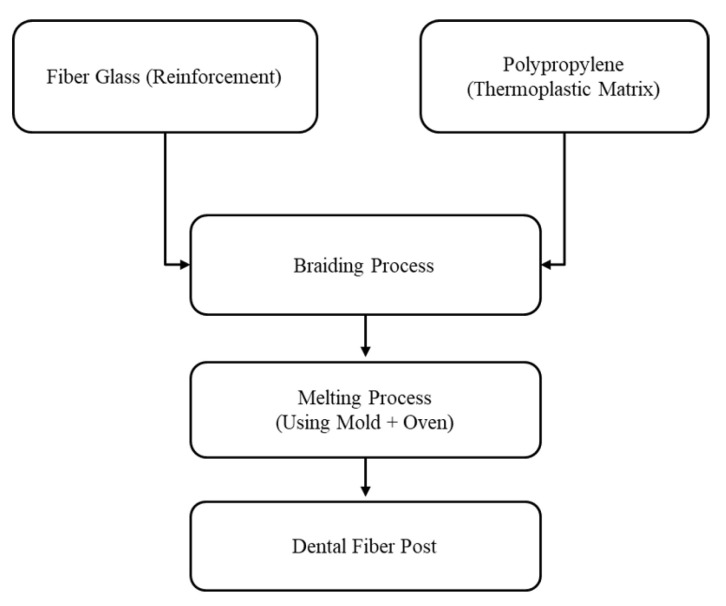
Flowchart for the process used in fabricating the FRP.

A flowchart summarizing the fiber post fabrication process is illustrated in Figure 1.

Different braiding constructions (sheath and core) were fabricated in the first stage using mixed sheaths (reinforcement and thermoplastic) and different cores.

In this stage, four categories of braided processes—according to their core type—were produced, with each of them possessing five different sheath structures.

The four different cores were: (A) reinforcement fibers (glass fiber), (B) thermoplastic fibers (polypropylene), (C) mixed fibers (glass fiber and polypropylene), and (D) full braided core of both reinforcement and thermoplastic fibers.

For core (D), all of the produced braids with the last three cores (A, B, and C) were -as a whole- used as a core of a fully thermoplastic sheath which then produced double sheath braids in a trial to enhance the thermoplastic coverage of the final post. Table 5 shows the different used setups for the five different five with their four different cores.

A second stage of producing braids was executed using the other type of glass fiber yarns (E-glass and S-glass) with a special silane to enhance the adherence with the thermoplastic fibers and to test the different effects of both types of glass fiber (with silane and with no silane) on the final fiber post properties.

The setup of the braid’s sheath in this stage was consisting of 16 working spindles aligned with the same three first cores (A, B, and C) used in the first stage.

This setup was used twice, once with the E-glass fiber and the other with the S-glass fiber, as shown in Table 6.

**Figure 2:**
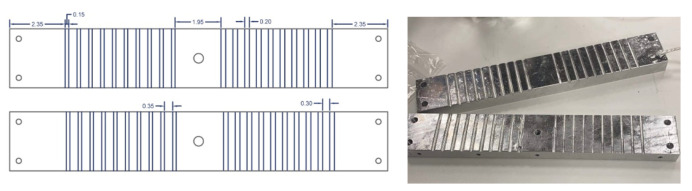
Custom-made aluminum mold.

The produced braids were then placed in an aluminum mold (Figure 2) after being cut from the braiding machine. The mold was grooved with two different diameters (1.5–2 mm) with 10 grooves per diameter. Two similar molds were used throughout the melting process to accelerate the production. These two molds with the braided processed inside were placed in a digital oven at the melting temperature of the used thermoplastic (PP: 165 °C ± 5) for almost 40 min (±5 min) in order to obtain the final shape of the post.

**Figure 3:**
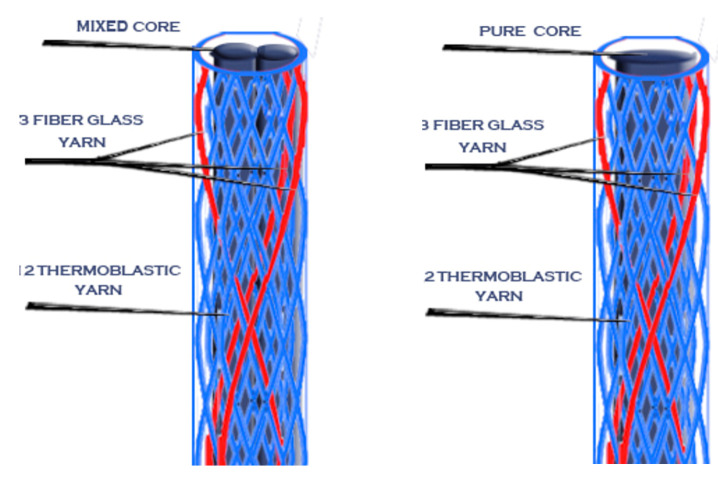
Sample 1 with different cores.

**Figure 4:**
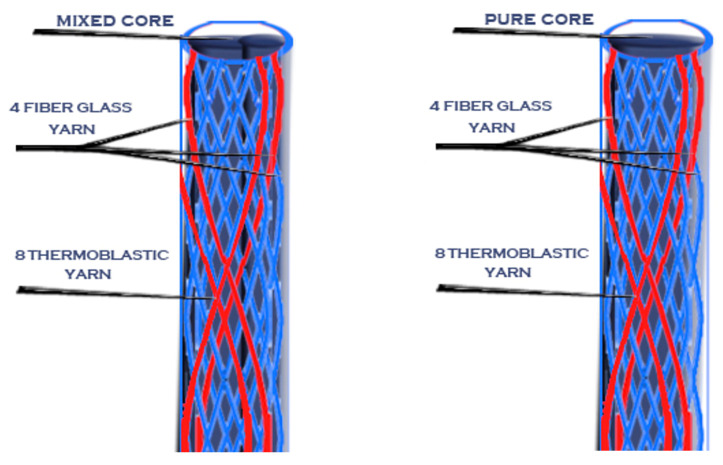
Sample 2 with different cores.

**Figure 5:**
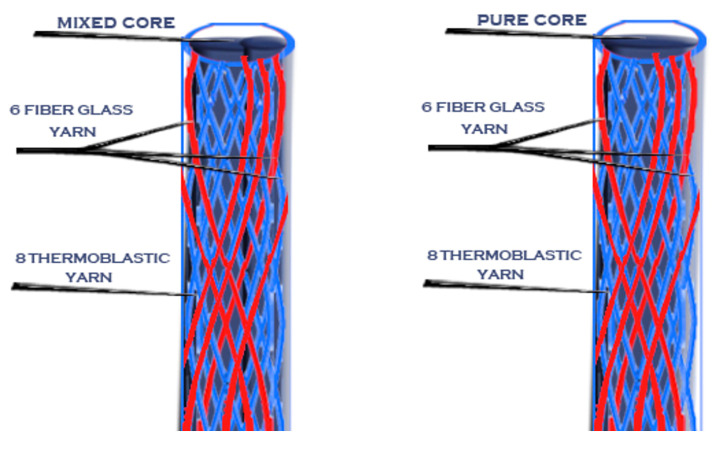
Sample 3 with different cores.

**Figure 6:**
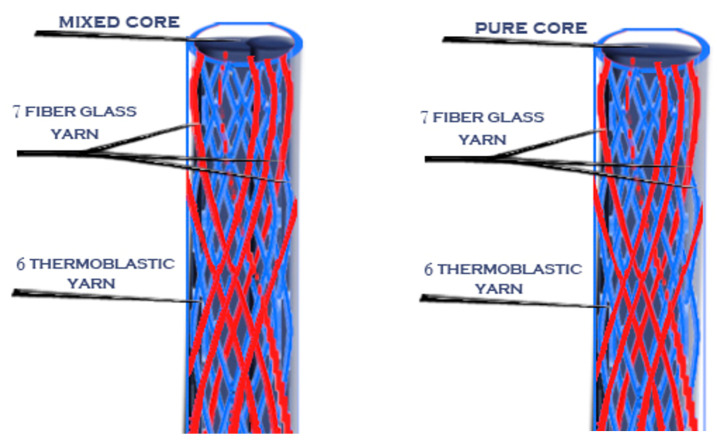
Sample 4 with different cores.

**Figure 7:**
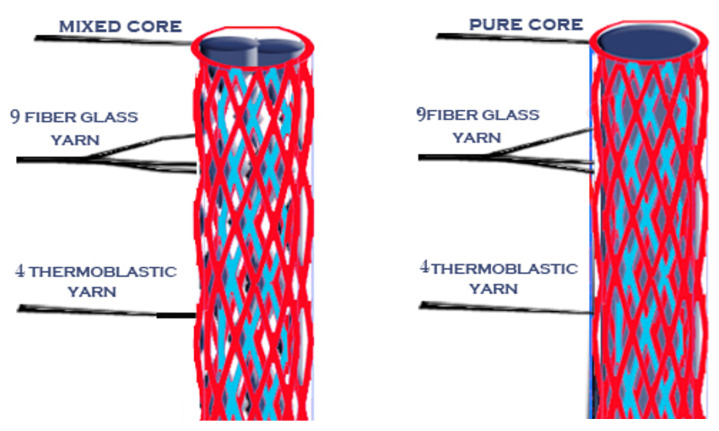
Sample 5 with different cores.

**Figure 8:**
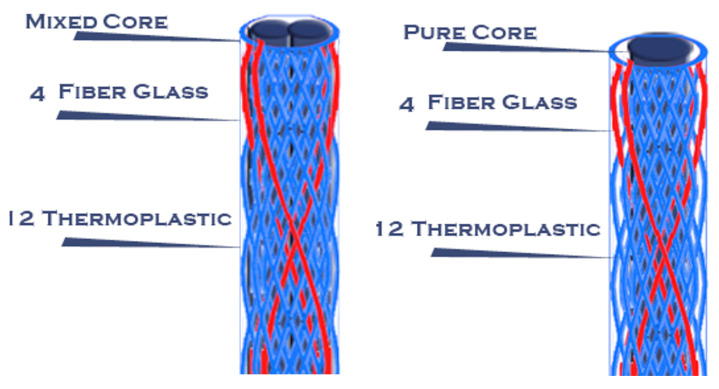
Samples 6 and 7 with different cores.

Then, after the mold was cooled, the posts were removed from it using a very thin needle. Novel posts with different braiding structures and core types are presented in the following 3D figures (Figure 3, Figure 4, Figure 5, Figure 6, Figure 7 and Figure 8). Later, in the second stage, these samples were covered with a PP sheath consisting of 16 yarns (300 Denier each).

### 2.3. Testing

**Figure 9:**
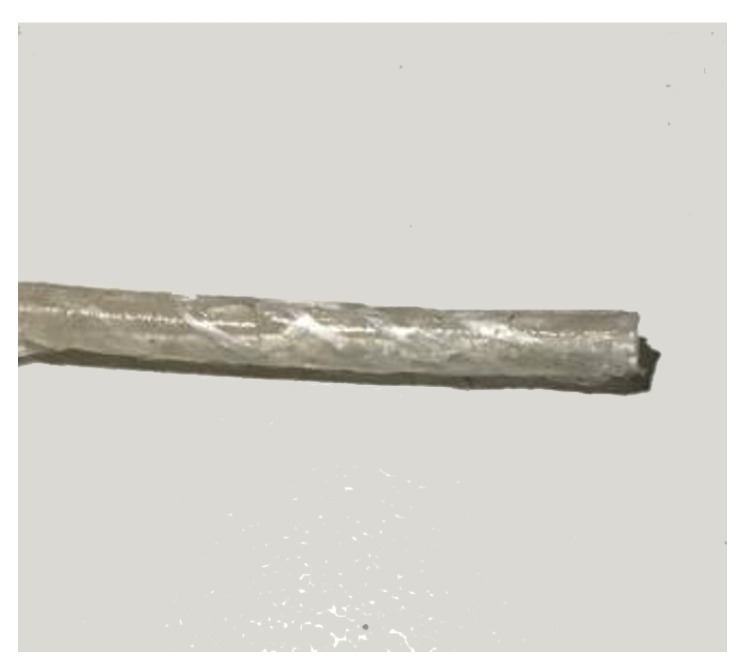
Real photo of the resulting post.

Samples 6 and 7 in Figure 8 represent the last stage using the treated glass fiber yarns with silane (S-glass and E-glass). All of the aboce figures were drawn to illustrate the used braiding’s structures and the ratio between the PP yarns to the glass fiber yarns within the sheath and core in the formed posts. Figure 9 shows the real post that occurred after being melted and removed from the mold for completion to remove any growths using soft emery or similar tools and that may have been tapered manually prior to being implanted in a real tooth if needed.

Non-destructive tests were performed on all of the braided products an using Olympus Epoch 650 device (OLYMPUS Corporation, Tokyo, Japan) to measure the Young’s modulus and the shear modulus of the novel FRC post specimens by measuring the P-wave and the S-wave velocities three times per each sample and then calculating their means and standard deviations [27].

Using the following formulas, both Young’s modulus and shear modulus were calculated:

(1) $$C_{p}=\sqrt[{}]{\frac{E(1-v)}{p(1+v)(1-2v)}}$$

(2) $$C_{s}=\sqrt[{}]{\frac{E}{2p(1+v)}}=\sqrt[{}]{\frac{G}{p}}$$

where $C_{p}$ is the p velocity, $C_{s}$ is the shear velocity, *E* is the Young’s modulus, *G* is the shear modulus, *v* is Poisson’s ratio, and rho is the post density.

Fifty different FRC posts were tested using the ultrasonic test to identify each post’s Young’s and shear modulus [28].

**Figure 10:**
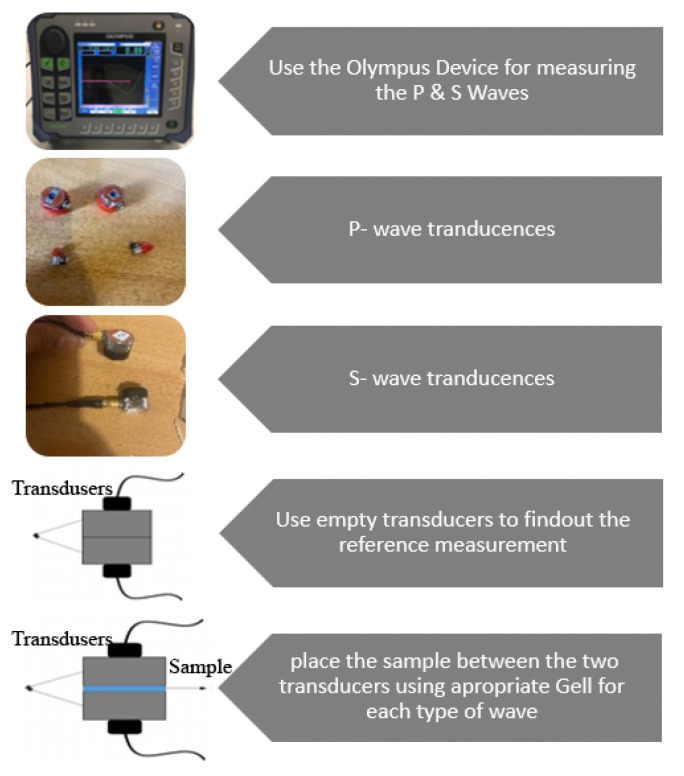
Flowchart representing a scheme with the connections and sensors, and a representation of the experimental measurements and procedure.

Figure 10 shows a flowchart with a scheme and pictures of the device, connections, sensors (with typology), and frequency, as well as a representation of the experimental measurements and procedure used in the testing phase.

## 3. Results

All of the tested samples showed Young’s modulus’ values ranging from 6.78 GPa to 30.19 GPa and shear modulus’ values ranging from 2535.61 MPa to 11,076.18 MPa.

The highest modulus was for sample number 7, which is a braided product consisting of a sheath of sized S-glass fiber (with silane) braided with polypropylene yarns (25%:75%) and a mixed core, while the lowest one was for sample number 5 covered, which is a braided product consisting of a sheath of E-glass fiber with polypropylene (75%:25%) and a PP core, which was covered with a full PP sheath. The remaining samples ranged between these two values.

The recommended sample was sample number 6, which is a braided product consisting of a sheath of sized E-glass fiber (with silane) with polypropylene (25%:75%) and a GF core with a Young’s modulus’ value of 17.17 GPa and shear Modulus of 6217 MPa, as shown in Table 7. The reason for this is that its Young’s modules is the closest to the that of the human dentin (18 GPa).

## 4. Discussion

**Figure 11:**
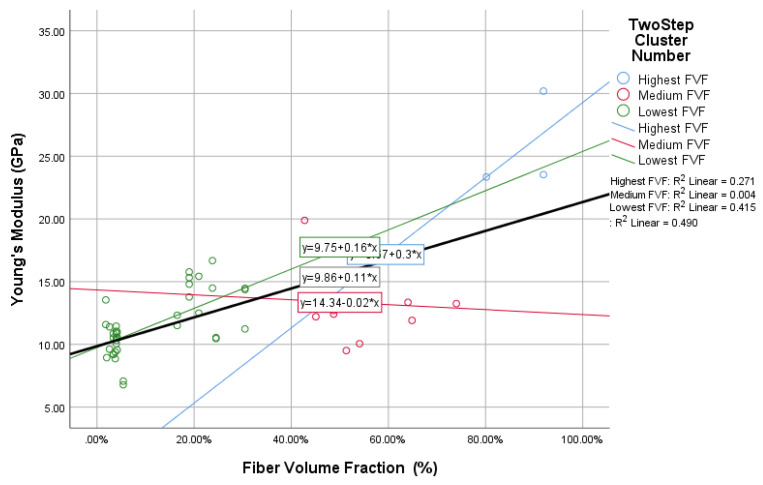
The relationship between fiber volume fraction and Young’s modulus.

**Figure 12:**
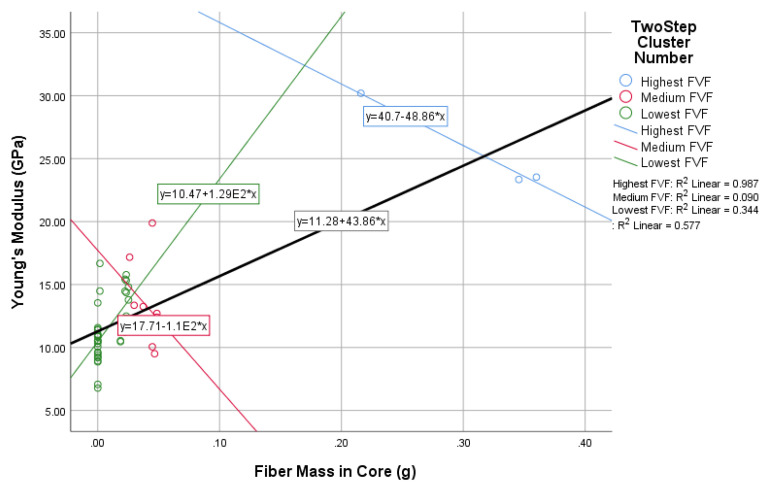
The relationship between fiber mass in the core and Young’s modulus.

**Figure 13:**
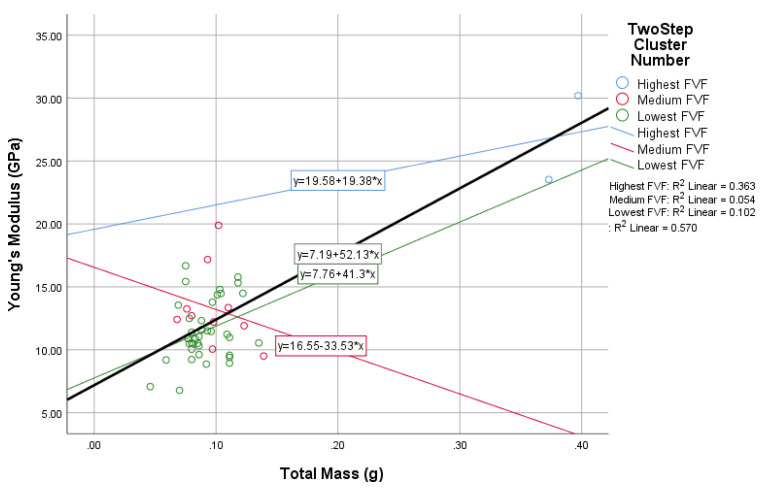
The relationship between fiber mass and Young’s modulus.

**Figure 14:**
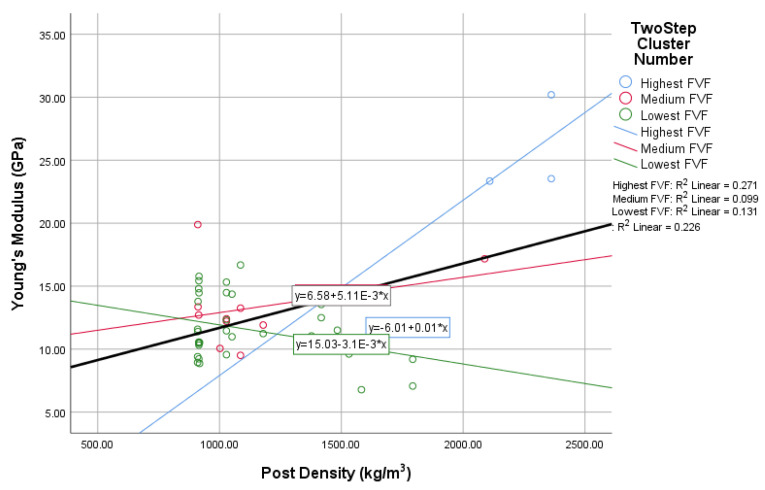
The relationship between post density and Young’s modulus.

**Figure 15:**
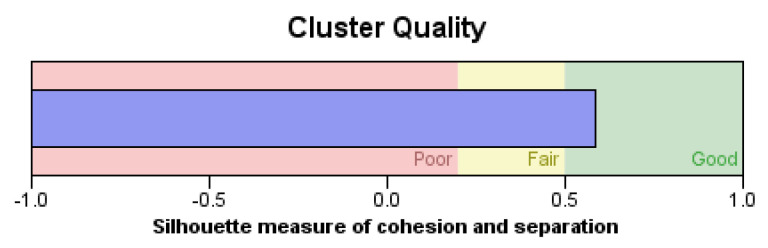
Silhouette measure of cohesion and separation.

**Figure 16:**
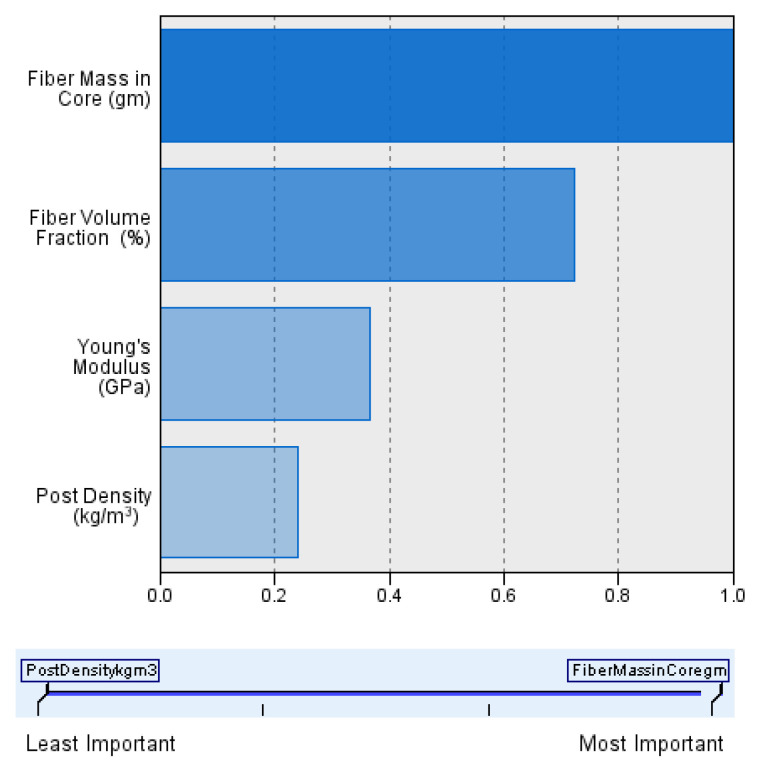
Importance predictor measurement.

In this study, the novel fabricated glass-fiber-reinforced posts were affected by the core type, fiber volume fraction (FVF), and post density as shown in Figure 11, Figure 12, Figure 13 and Figure 14. This fact is supported by the results of the two-means, cluster which revealed that all of the samples were divided into three main clusters (first: highest FVF, second: medium FVF, and third: lowest FVF) with a good silhouette measure of cohesion and separation (Figure 15), and the highest predictor importance regards the fiber mass in the core (Figure 16).

Accordingly, the main difference between each cluster was their percentage of FVF, which, in turn, depended mostly on the fiber mass in the core in relation to the fact that the highest contributor in the post-composition is the core mass.

Glass fiber and mixed cores samples (6-, 7-, and 1-covered) showed significantly higher shear modulus and Young’s modulus than those of the polypropylene cores. The core mass in the structure of the novel fabricated posts represents almost 70% of the total mass of the whole post.

These findings support the fact that the higher the FVF in the composite post, the higher the posts’ Young’s modulus becomes until reaching a certain level where the Young’s modulus value then significantly drops down due to the lack of the resin (thermoplastic) covering the fiberglass yarns to solidify the resultant post and form the post’s final shape.

Figure 11 shows that there is a direct relationship between the FVF percentage and the Young’s modulus of the resulted posts—and, consequently, the shear modulus—represented in a linear equation with an R² value of 0.49 for the whole samples and a *p*-value of 0.7, while R² values in the subgroups—represented in the three resulting clusters—were 0.27 and 0.42 for the first and third clusters, respectively, and a *p*-value of 0.52 and −25, respectively. The linear relationship in the second cluster was not as significant as that of the other two clusters, with an R² value of 0.004; however, there was still an obvious relation represented by the *p*-value of 0.27. These results show a strong relationship between the FVF and the resultant Young’s modulus.

Figure 12 shows a directly proportional relationship between the core type (represented in the core fiber mass) and the Young’s modulus of the final post. This relationship is demonstrated by the linear equation with an R² value of 0.58 for the whole samples and a *p*-value of 0.76, while R² values for the three different clusters were 0.99, 0.09, and 0.34, and the *p*-values were −0.99, 0.40, and −0.32.

A linear relationship was noticed to exist between the fiber mass in the core and the resulting Young’s modulus; two clusters showed an inverse relationship due to two following main reasons: the extra high FVF in the first cluster (92%) and the extra low FVF in the third cluster (20%).

Additionally, a strong linear direct relationship between the fiber mass in the core and the resulting Young’s modulus could also be concluded within an average percentage of the FVF.

Figure 13 supports the results inferred from the two previous figures (Figure 11 and Figure 12) as it show that the fiber mass in the whole post is directly proportional to the posts’ Young’s modulus as well. However, the third cluster has different results due to the due to the lack of FVF.

This direct relationship could be inferred from the linear equation with an R² value of 0.57 for the whole samples and a *p*-value of 0.76, while the R² value for the three clusters were 0.36, 0.05, and 0.10 with *p*-values of 0.60, 0.16, and −0.30.

**Figure 17:**
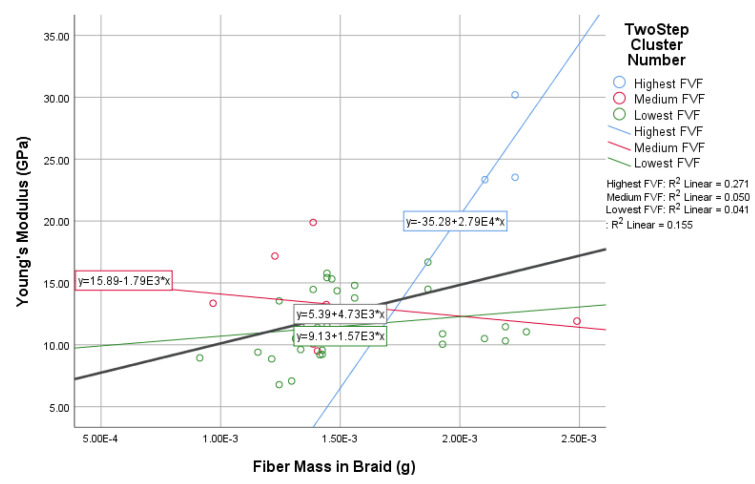
The relationship between fiber mass in the braid’s sheath and Young’s modulus.

The fiber post density also showed a considerable effect on the fiber posts’ final properties, especially the posts’ Young’s modulus, as shown in Figure 17. This relationship is represented in a linear equation with an R² value of 0.23 and a *p*-value of 0.48. In contrast, the R² values for the three resulted clusters were 0.27, 0.09, and 0.13 with *p*-values of 0.52, −0.20, and 0.17.

In conclusion, a directly proportional relationship was found between the FVF value, the glass fiber mass in the core, and the resultant Young’s modulus values to a certain extent and then Young’s modulus values dropped down dramatically (as shown in the above Figure 11, Figure 12, Figure 13 and Figure 14). This conclusion can be explained by the fact that the initial Young’s modulus of the polypropylene is only 1.38 GPa, while the Young’s modulus of the glass fiber that is responsible for the FVF and fiber mass values is about 72 GPa. Thus, the more the amount of glass fiber in the post, the higher the resultant Young’s modulus, which allows complete coverage of the glass fibers with thermoplastic fibers (polypropylene) acting as the matrix part (resin) of the composite in the current study.

In addition, the higher the fiber mass in the core, the higher the fiber mass in the whole post, as the core mass is almost 70% of the whole post mass due to the braiding structures, yarn counts, and braiding parameters used in the current study.

Previous studies have shown that the glass fiber volume fraction affects the posts’ final properties in terms of Young’s modulus values [29,30]. Additionally, studies have shown that the Young’s modulus of FRC is affected by the ratio of the glass fiber to the matrix resin [31], but previous studies have not mentioned the effect of the core type—represented by the fiber amount and allocation within the post—on the fiber post’s physical properties, which was proved to be a significant factor (R² = 0.49) affecting the final post rigidity represented by the Young’s modulus value, as shown in Figure 11.

There were no significant differences in the Young’s Modulus values (R² linear = 0.16) among the whole samples with different cores and different percentages of glass fiber yarns in their braid’s sheath (represented in the mass in the braid), as shown in Figure 17. In addition, a weak relationship between both fiber mass in the braid and Young’s modulus was indicated by the *p*-value of (0.4). Meanwhile, the R² value for the three resulting clusters were 0.27, 0.05, and 0.04 with *p*-values of 0.52, 0.25, and −0.02.The reason could be inferred from the structure of the braid (post) whose core occupies 70% of the post with the sheath occupying only 30%.

This suggests that the main characteristics of the final post are almost defined by the core type, but the sheath plays a very important role in holding and supporting the post’s core tightly and preventing or at least decreasing the gaps in the final structure of the post in order to avoid voids and cracks in the final produced post after the melting process.

On the other hand, the flexural strength was affected by post diameter and homogeny [32]. Literature reviews have shown that stresses normally develop at the interface between the fibers and the matrix and propagate along the surfaces of the fibers when the posts are loaded [33,34,35,36,37,38,39,40]. Some researchers have noted that internal defects, such as voids, cracks, or micro-bubbles, inside the prefabricated glass fiber post weaken the final post [41,42]. Previous reports indicated that concentrated stress leads to failure of adhesion at the interface between the fiber and the matrix resin, resulting in micro-cracks [43,44,45,46].

In the present study, there were some voids at the interface between the glass fiber and the matrix resin due to the used basic method. These voids affect the final posts’ properties negatively by weakening their mechanical performance, which is represented by low values of both Young’s and shear moduli for some samples, as shown in Table 7. These voids are supposed to disappear or even decrease using a more automated production method.

## 5. Conclusions

In this study, the results of a new method of fabricating dental composites based on braiding thermoplastic yarns with glass fiber yarns were presented and experimentally validated. The braiding process showed a feasible method of fabricating dental fiber posts, while composite formation was achieved using melting process of the thermoplastic yarns, which demonstrated a superior performance when used as a matrix in the FRC posts. The results are consistent with the tested conceptual hypotheses: the braiding procedure provided a good solution for fabricating dental fiber posts, thermoplastic filaments were used as a matrix of reinforced composites for dental fiber posts in an ex vivo application, the fiber volume fraction was directly proportional to the final post modulus, and the fiber material’s native Young’s modulus is correlated to the post’s Young’s modulus and shear modulus.

Although the braiding technique of thermoplastic fibers with glass fibers improved the mechanical compatibility of the resulting posts (ex vivo), considerable variations could be found in the calculated modulus values of the studied novel fabricated posts due to the basic manual manufacturing process used in this study, which led to some irregularities in the produced posts.

In addition, the flexural strength of the prefabricated glass fiber post demonstrated a tendency to decrease with the reduction in the fiber volume fraction, especially in the core, which is represented by the core type in the used setups.

The R² value per each cluster defined some relationships, but it was not significant for all relations because some of the clusters that contained a small number of samples—especially cluster one—which also made the predictive power of regression a preliminary one in terms of the clusters. Moreover, regarding the outliers, some of the predictions may be different if more samples are considered.

All of the newly fabricated samples were tested using the ultrasound testing procedure and showed Young’s modulus ranges of from 6.78 GPa to 30.19 GPa and shear modulus ranges of from 2535.61 MPa to 11,076.18 MPa.

A directly proportional relationship among the fiber volume fraction values, glass fiber mass as a setting parameter, and the resulting Young’s modulus value was determined from the results.

Future experimental studies must be carried out in order to test the mechanical performance using mechanical testing procedures and by testing endodontically treated human teeth with the post samples inside. These future studies will help in indicating and enhancing the performance of the newly fabricated posts using the novel method of the current study.

## 6. Patents

“BRAIDED FIBER REINFORCED COMPOSITE POST”, IPR in Granada university: 847.

## Figures and Tables

**Table 1:** E-glass fiber yarns specs [1].

Product	ECE225
Glass Type	E
Filament Diameter	microns 7
Binder	622
Bobbin	8542
Plies	1/0
Nominal Yield, yd/lb	22,500
Tex, g/1000 m	22
Tex tolerance +/	1.2
Nominal Solids %	1.4
Solids Tolerance +/−	0.25
Nominal Twist TPI (TPM)	0.5Z (Z20)
Twist Tolerance +/− TPI (TPM)	0.15 (6)
Max. Broken Filaments	10
Minimum Tensile, lb (N)	2.4 (10.7)
Average Bare Glass Tensile, lb (N)	3.48QW (15.5)
Approximate Yarn Diameter, in (mm)	0.0065 (0.165)

**Table 2:** S-glass fiber-sized yarns specs [1].

Product	SCG75
Glass Type	S–2 GLASS
Filament Diameter	9
Binder	561
Bobbin	7636
Plies	1/0
Nominal Yield, yd/lb	7295
Tex, g/1000 m	68
Tex tolerance +/	6.2
Nominal Solids %	1.17
Solids Tolerance +/−	0.26
Nominal Twist TPI (TPM)	1.0Z (Z40)
Twist Tolerance +/− TPI (TPM)	0.3 (12)
Max. Broken Filaments	9
Minimum Tensile, lb (N)	7.1(31.6)
Average Bare Glass Tensile, lb (N)	10.8 (48)
Approximate Yarn Diameter, in (mm)	0.0076 (0.192)

**Table 3:** E-glass fiber-sized yarns specs [1].

Product	ECDE75
Glass Type	E
Filament Diameter	6
Binder	561
Bobbin	8571
Plies	1/0
Nominal Yield, yd/lb	7500
Tex, g/1000 m	66.1
Tex tolerance +/	4.3
Nominal Solids %	1.42
Solids Tolerance +/−	0.17
Nominal Twist TPI (TPM)	0.7Z (Z28)
Twist Tolerance +/− TPI (TPM)	0.21 (8)
Max. Broken Filaments	10
Minimum Tensile, lb (N)	5.7 (25.4)
Average Bare Glass Tensile, lb (N)	9.6 (43)
Approximate Yarn Diameter, in (mm)	0.106 (0.269)

**Table 4:** Polypropylene yarns specs [24].

Yarns Type	Polypropylene (PP)
Count	300 Denier
Melting point	180 °C
Young’s Modulus (GPa)	1.38
Tensile strength (MPa)	34
Approximate Yarn Diameter, in (mm)	0.0045

**Table 5:** Different setups for the braiding structure using the E-glass fiber.

Sample No.	TP:GF:Mixed Spindles	TP:GF:Mixed Count (Den)	Total Spindles	Total Counts (Den)	TP:GF %	Core (A)	Core (B)	Core (C)	Double Sheath (D)
1	12:3:0	300:400:0	15	4800	75:25	6 GF yarns each, 2800 = 16,800 Denier	Two yarns of PET each, 8400 = 16,800 Denier	3 GF yarns each 2800, = 8400 Denier + one single yarn of PET, 8400 Denier	each processed braid was covered with a secondary fully thermoplastic (TP) sheath of 16 spindles; each is 300 Denier
2	8:4:1	300:400:500	13	4500	60:40				
3	8:6:0	300:400:0	14	4800	50:50				
4	6:7:0	300:400:0	13	4600	40:60				
5	4:9:0	300:400:0	13	4800	25:75				

**Table 6:** Different setups for the braiding structure using the sized E- and S -glass fibers.

Sample No.	TP:GF Spindles	TP:GF Count (Den)	GF Type	TP:GF %	Total Spindles	Total Counts (Den)	Core(A)	Core(B)	Core(C)
**6**	12:4	300:400	E-glass fiber	60:40	16	5000	6 GF yarns each, 2800 = 16,800 Denier	Two yarns of PET each, 8400 = 16,800 Denier	3 GF yarns each, 2800 = 8400 Denier + one single yarn of PET, 8400 Denier
**7**	12:4	300:400	S-glass fiber	60:40	16	5000			

**Table 7:** The highest and lowest groups regarding the posts’ Young’s modulus.

Sample Code/Core	Sample No.	Young’s Modulus GPa Mean (SD)	Shear Modulus MPa Mean (SD)	Core Mass g Mean (SD)	Length in mm Mean (SD)	Mass in g Mean (SD)	Post Density in kg/m${}^{3}$ Mean (SD)	Fiber Volume Fraction % Mean (SD)
S-GF/SS6/MIX	7	30.19 (1.16)	10,503.68 (404.82)	0.22 (0.01)	25 (0.20)	0.40 (0.02)	2361.81 (0)	92% (0)
E-GF/SS6/GF	6	23.53 (0.50)	8181.80 (57.20)	0.36 (0.01)	24 (0.18)	0.37 (0.01)	2361.81 (0)	92% (0)
S-GF/SS6/GF	7	23.34 (0.41)	8113.89 (34.97)	0.35 (0.01)	15 (0.10)	0.18 (0.01)	2109.12 (0)	80% (0)
CSS1/GF	1 covered	19.89 (0.33)	7422.85 (121.35)	0.04 (0.00)	25 (0.20)	0.10 (0.01)	910.73 (0)	43% (0)
E-GF/SS6/GF	6	17.17 (1.24)	6217.00 (448.14)	0.03 (0.00)	25 (0.16)	0.09 (0.01)	2088.16 (0)	65% (0)
E-GF/SS6/MIX	6	16.68 (0.71)	6354.06 (268.88)	0.00 (0)	20 (0.20)	0.08 (0.00)	1086.06 (0)	24% (0)
CSS3/PP	3 covered	9.40 (0.47)	3500.29 (174.52)	0.00 (0)	23 (0.17)	0.11 (0.01)	910.00 (0)	4% (0)
SS2/PP	2	9.22 (0.14)	3322.72 (48.82)	0.00 (0)	26 (0.23)	0.08 (0.00)	915.03 (0)	3% (0)
SS2/PP	2	9.19 (0.41)	3293.20 (146.80)	0.00 (0)	17 (0.10)	0.06 (0.01)	1793.04 (0)	3% (0)
SS1/PP	1	8.94 (0.32)	3260.44 (116.60)	0.00 (0)	24 (0.18)	0.11 (0.01)	910.00 (0)	2% (0)
CSS3/PP	3 covered	8.86 (0.07)	3219.48 (23.93)	0.00 (0)	25 (0.20)	0.09 (0.01)	917.99 (0)	4% (0)
CSS5/PP	5 covered	7.07 (0.12)	2641.29 (44.60)	0.00 (0)	14 (0.12)	0.05 (0.01)	1793.04 (0)	5% (0)
CSS5/PP	5 covered	6.78 (0.26)	2535.61 (98.61)	0.00 (0)	24 (0.20)	0.07 (0.01)	1582.42 (0)	5% (0)

## Data Availability

Data sharing not applicable.

## Abbreviations

The following abbreviations are used in this manuscript:

FRC	Fiber-reinforced composite
FVF	Fiber volume fraction
PP	Polypropylene
GF	Glass fiber
TP	Thermoplastic
Den	Denier
Cs	Shear velocity
Cp	P-velocity
MPa	Mega Pascal
GPa	Giga Pascal
SD	Standard deviation
